# Effect of Surface Scattering of Electrons on Ratios of Optical Absorption and Scattering to Extinction of Gold Nanoshell

**DOI:** 10.1186/s11671-018-2670-7

**Published:** 2018-09-25

**Authors:** Yiyang Ye, T. P. Chen, Zhen Liu, Xu Yuan

**Affiliations:** 1 School of Electrical and Electronic Engineering, Nanyang Technological University, Singapore, 639798 Singapore; 2 School of Materials and Energy, Guangdong University of Technology, Guangzhou, 510006 People’s Republic of China; 3 School of Materials Science and Engineering, Nanyang Technological University, Singapore, 639977 Singapore

**Keywords:** Nanoshell, Surface scattering, Light absorption, Light scattering

## Abstract

Gold nanoshell’s high light scattering and absorption at its resonance wavelength have found applications in biomedical imaging and photothermal therapy. However, at nanoscale, metallic material’s dielectric function is affected by nanoparticle’s size, mainly via a mechanism called surface scattering of conduction electrons. In this work, the effect of surface scattering of electrons on the ratios of optical absorption and scattering to extinction (which is the sum of the absorption and scattering) of gold nanoshell is investigated. Simulation results for several shell thicknesses are compared. It is found that the electrons’ surface scattering increases the optical absorption ratio, and the thinner the shell thickness, the larger the increase in the difference of the absorption ratio between the situations with and without the surface scattering considered. The increase of absorption ratio is then verified by comparing simulation results to experimental measurements for three nanoshells. The parameters of the simulations to fit the experimental measurements show that the damping of conduction electrons in metallic shell geometry is larger than that predicted by the billiard scattering model.

**Electronic supplementary material:**

The online version of this article (10.1186/s11671-018-2670-7) contains supplementary material, which is available to authorized users.

## Background

Gold nanoshell is composed of a dielectric core, which may be silica or *Au*_2_*S* [1, 2], and a concentric shell of gold. Due to gold’s biocompatibility [3, 4], facile conjugation of antibodies and targeting moieties to gold shell’s surface [5], tunability of its resonant wavelength to the near infrared region [2, 6], and a region called the biological water window where the tissue transmissivity is the highest [7], gold nanoshell’s enhanced light scattering and absorption have found applications in biomedical imaging and photothermal therapy [8, 9]. Mie theory’s extension for core-shell structure can be employed to calculate a single gold nanoshell’s optical absorption and scattering cross sections [10], and the sum of these two gives its extinction cross section. Since the thickness of gold nanoshell is usually smaller than or comparable to electron’s mean free path in bulk gold, which is about 37.7 nm [11], electrons in gold shell go through more collisions per unit time (extra collisions caused by scattering of conduction electrons from shell surface) than they do in bulk gold [12, 13]. Surface scattering of conduction electrons has been reported to cause broadening of resonance peak, which was verified by fitting of measured and calculated spectra [6, 14–16], and reduction in the absolute values of both scattering and absorption of a single nanoshell which was demonstrated by theoretical calculations [17–19]. However, for scattering-based biomedical imaging applications [9, 20, 21], where metallic nanoparticles or fluorescent materials are attached to target tissue or cells, if it is desired to only image and not thermally damage the tissue or cells under investigation, it is important that the attached nanoparticle has high ratio in scattering and low ratio in absorption at the desired wavelength. The reason why ratios of scattering and absorption but not their absolute values are of concern is that decrease in absolute values of scattering and absorption can be compensated by having more particles attached to the target tissue or cells. Application of metallic nanoparticle’s resonant light scattering in transparent projection screen [22–25], and in photovoltaics [26–30], also requires simultaneous high scattering and low absorption ratios at the desired wavelength range. What is more, gold metamaterials also require gold to be of the form of thin film, which can achieve high optical absorption as light absorber [31, 32], or high transmittance as transparent conducting film [33–36], and thus, conduction electrons’ surface scattering effect plays a role too. Therefore, it may give some guidance on designing nanoscale gold-related structure to investigate the effect of surface scattering of electrons on the ratios of optical absorption and scattering to extinction for gold nanoshells.

In this work, simulations are first conducted to study the effect of surface scattering of electrons on the ratios of optical absorption and scattering to extinction of gold nanoshells by considering the situations with and without the surface scattering. It is shown that the electrons’ surface scattering increases the optical absorption ratio and therefore decreases the light scattering ratio, and the thinner the shell thickness, the larger the increase in the optical absorption ratio. The increased absorption is then verified experimentally for three samples by comparing their measured and simulated absorption as well as extinction spectra.

The simulation and experimental results will be shown first in the “Results and Discussion” section, and then, detailed method of optical measurements of extinction and absorption is provided in the “Methods/Experimental” section, to avoid unnecessary confusion caused by the descriptions of optical measurements.

## Results and Discussion

Gold nanoshells with four different shell thicknesses but same core diameter are studied by simulation. The gold nanoshells include (80-nm-diameter silica core)@(15-nm-thick gold shell), (80-nm-diameter silica core)@(25-nm-thick gold shell), (80-nm-diameter silica core)@(35-nm-thick gold shell), and (80-nm-diameter silica core)@(45-nm-thick gold shell).

After interaction of parallel incident rays of light with a single nanoparticle, apart from those directly transmitted (propagating in the original direction of the incident light), light is either absorbed or scattered, and the sum of these two is referred to as extinction [37]. The scattering, absorption, and extinction, quantized in terms of cross sections, which can be intuitively perceived as the area amount of light removed from the path of incident light due to scattering, absorption, or extinction respectively, can be calculated by Mie theory’s extension for core-shell structure [10]. However, it is more natural to normalize the cross sections to the nanoparticle’s geometric cross section, *πR*^2^, where *R* is the outer radius of a core-shell structure, for the purpose of comparison between different structures, and the ratio of *j* cross section (*j*=absorption, scattering, or extinction) to the geometric cross section is termed as *j* efficiency.

**Fig. 1:**
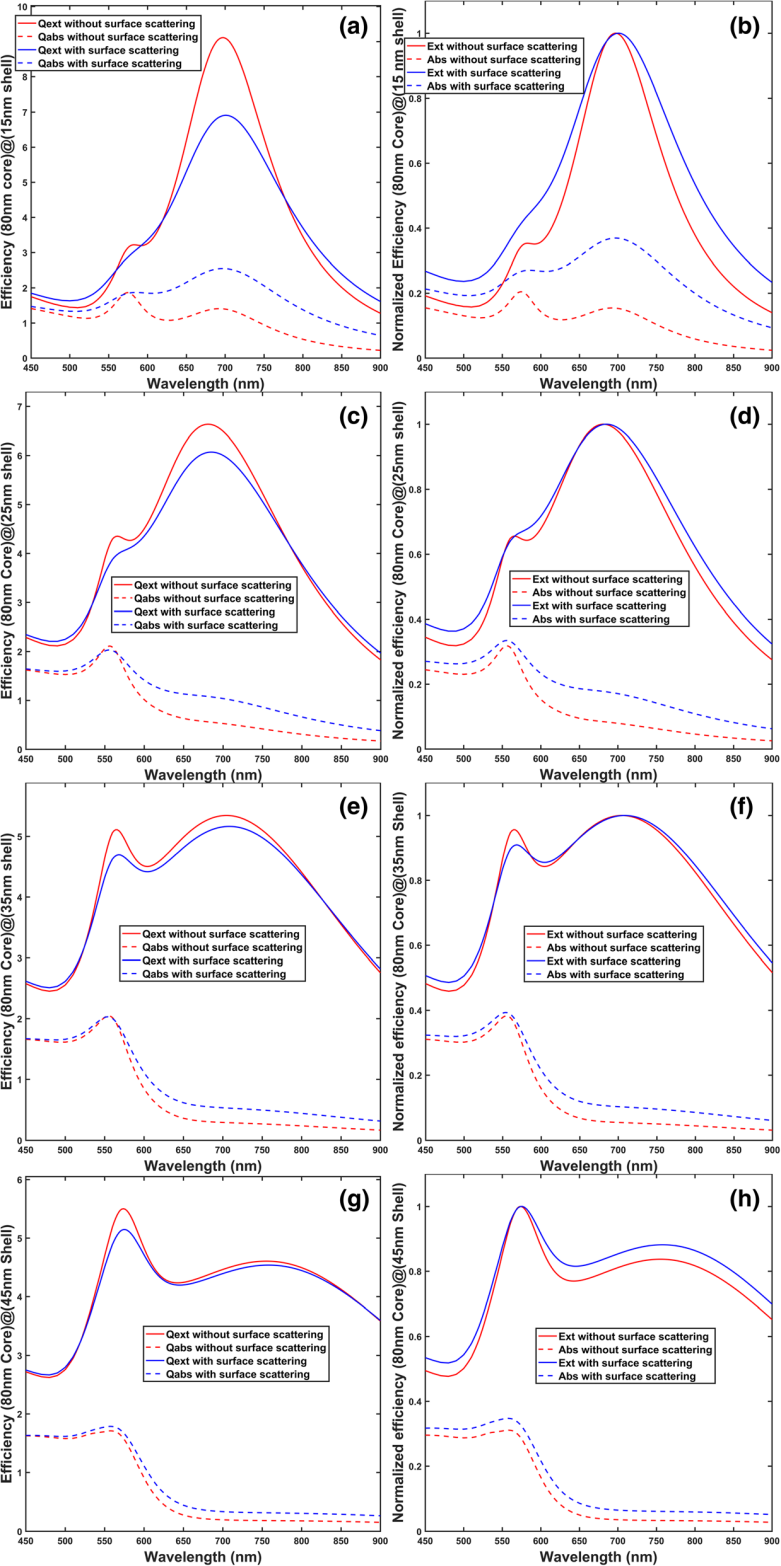
Calculated extinction and absorption efficiencies with and without consideration of surface scattering of conduction electrons, where *Q*_ext_ (Ext) stands for extinction efficiency (normalized extinction) and *Q*_abs_ (Abs) stands for absorption efficiency (normalized absorption). The scattering efficiency (normalized scattering) is the difference between the *Q*_ext_ (Ext) and *Q*_abs_ (Abs). All calculations are carried out by Mie theory, with silica and surrounding medium’s indices assumed to be 1.45 and 1.5 respectively. Gold’s dielectric constants without surface scattering are from reference [38], while those with surface scattering are given by Eqs. (1)~(3)*.* **a** and **b** are for (80-nm-diameter silica core)@(15-nm-thick gold shell). **c** and **d** are for (80-nm-diameter silica core)@(25-nm-thick gold shell). **e** and **f** are for (80-nm-diameter silica core)@(35-nm-thick gold shell). **g** and **h** are for (80-nm-diameter silica core)@(45-nm-thick gold shell). The left column, i.e., **a**, **c**, **e**, and **g**, is the corresponding efficiencies as calculated by Mie theory. The right column, i.e., **b**, **d**, and **f**, is the efficiencies normalized to the dipolar resonance peak (the resonance peak between 700 and 800 nm), and **h** the efficiencies normalized to the quadrupolar resonance peak (the peak between 550 and 600 nm)

The extinction and absorption efficiencies without consideration of surface scattering effect are calculated by using bulk gold’s dielectric function [38] as input to the Mie theory, and they are shown as red lines (solid or dashed) in Fig. 1. To take into account the effect of surface scattering, it is assumed that the dielectric function of gold has a Drude model component to describe the behavior of free electrons [39], and an extra damping term *γ*_s_ contributed by surface scattering of conduction electrons is added to the bulk damping *γ*_b_ in the Drude term to give the corrected dielectric function *ε*_sh_ for gold shell [19]:

1 $$ {\varepsilon}_{\mathrm{s}\mathrm{h}}={\varepsilon}_{\mathrm{exp}}+\frac{\omega_{\mathrm{p}}^2}{\omega \left(\omega +i{\gamma}_{\mathrm{b}}\right)}-\frac{\omega_{\mathrm{p}}^2}{\omega \left[\omega +i\Big({\gamma}_{\mathrm{b}}+{\gamma}_{\mathrm{s}}\right]} $$

where *ε*_exp_ is gold’s bulk dielectric function from reference [38], *ω*_p_ is the plasma frequency of gold, *ω* is the frequency of the incident light, and *i* is the imaginary number. For the calculated efficiencies with surface scattering effect in Fig. 1 (blue lines, solid, or dashed), *ω*_p_ and γ_b_ are assumed to be 8.55 eV and 18.4 meV respectively [19]. And *γ*_s_ is given by [19]:

2 $$ {\gamma}_{\mathrm{s}}=\frac{v_{\mathrm{F}}}{L_{\mathrm{B}}} $$

where *v*_F_ is the Fermi velocity of electrons in gold and is equal to 1.40 × 10^6^m/s [19] and *L*_B_ is the effective mean free path of the electrons in the shell, derived by assuming billiard scattering model [13], in which the reflections of electrons from the two surfaces of shell are specular, and is given by

3 $$ {L}_B=\frac{4\left({r}_{\mathrm{o}}^3-{r}_{\mathrm{i}}^3\right)}{3\left({r}_{\mathrm{o}}^2+{r}_{\mathrm{i}}^2\right)} $$

where *r*_*o*_ and *r*_i_ are the outer and inner radius of the nanoshell respectively. The refractive indexes of the surrounding medium and silica core are assumed to be 1.5 and 1.45 respectively.

**Fig. 2:**
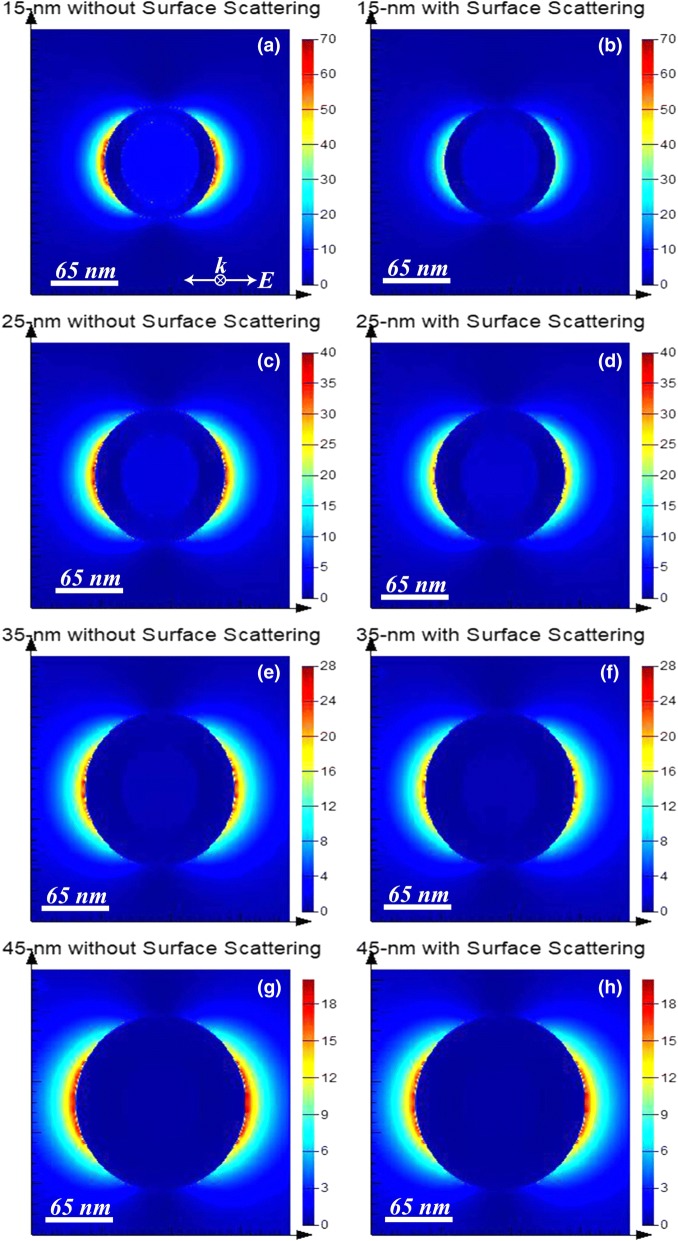
Square of near electric field amplitude |*E*|^2^ plots of the four structures shown in Fig. 1 at their corresponding dipolar resonance peak wavelengths. **a** and **b** are plotted for (80-nm-diameter silica core)@(15-nm-thick gold shell) at 700 nm. **c** and **d** are plotted for (80-nm-diameter silica core)@(25-nm-thick gold shell) at 684 nm. **e** and **f** are plotted for (80-nm-diameter silica core)@(35-nm-thick gold shell) at 706 nm. **g** and **h** are plotted for (80-nm-diameter silica core)@(45-nm-thick gold shell) at 756 nm. The left column, i.e., **a**, **c**, **e**, and **g**, shows |*E*|^2^ calculated with bulk gold’s dielectric constants from reference [38]. The right column, i.e., **b**, **d**, **f**, and **h**, shows |*E*|^2^ calculated with gold’s dielectric constants modified with surface scattering via Eqs. (1)~(3). The polarization and the propagating direction of the incident light are same for all figures and are shown in **a**. Simulation is conducted by the software “FDTD Solutions,” with the grid size of three-dimensional mesh override region being 1 nm

From the left column of Fig. 1, for the four shell thicknesses, after including the surface scattering effect, it is observed that both the extinction and absorption spectrums experience a broadening and that while the extinction spectrums decrease in magnitude, the absorption spectrums increase a lot at the dipolar resonance peak (the peak between 700 and 800 nm) and seem to not change at the quadrupolar resonance peak (the peak between 550 and 600 nm). The decrease in extinction efficiency magnitude and increase in absorption efficiency magnitude lead to an increase in the ratio of absorption to extinction, after inclusion of the surface scattering effect. This is confirmed by the right column of Fig. 1 where it is observed that the absorption increases (i.e., the blue dashed lines are above the red dashed lines) at both the dipolar and quadrupolar peak positions. Intuitively, the increase in absorption ratio after considering surface scattering effect becomes less significant with increasing shell thickness, as can be observed in (b), (d), (f), and (h) in Fig. 1. This is because the thicker the shell, the less the frequency of electrons’ collisions with the shell surfaces, i.e., surface scattering effect is reduced. The phenomenon is also confirmed by Table 1. For each shell thickness, the absorption ratio with (without) surface scattering, calculated by the ratio of the region under the blue (red) dashed curve to the region under the blue (red) solid curve, is tabulated in Table 1. To further investigate the mechanism behind the increase of absorption ratio, spatial distributions of square of near electric field amplitude |*E*|^2^ are plotted in Fig. 2. In Fig. 2, it can be observed that |*E*|^2^*s* calculated without surface scattering are larger than those with surface scattering, which may be explained in this way: assuming surface scattering takes effect, conduction electrons experience more collisions from shell surfaces as compared to those in bulk gold, so conduction electrons’ average oscillation amplitude is decreased, leading to reduced |*E*|^2^*s*. And since collisions of conduction electrons with shell surfaces contribute to energy loss as heat, the absorption ratio is increased after including surface scattering effect.

**Table 1:** Comparison of the absorption ratio between the calculations with and without the surface scattering effect of conduction electrons. The results are obtained from Fig. 1

Shell thickness	15 nm	25 nm	35 nm	45 nm
Absorption ratio *without* surface scattering effect	25.92%	20.52%	17.83%	16.41%
Absorption ratio *with* surface scattering effect	45.30%	28.72%	22.30%	19.49%
Relative increase of absorption ratio	74.77%	39.96%	25.07%	18.77%

**Fig. 3:**
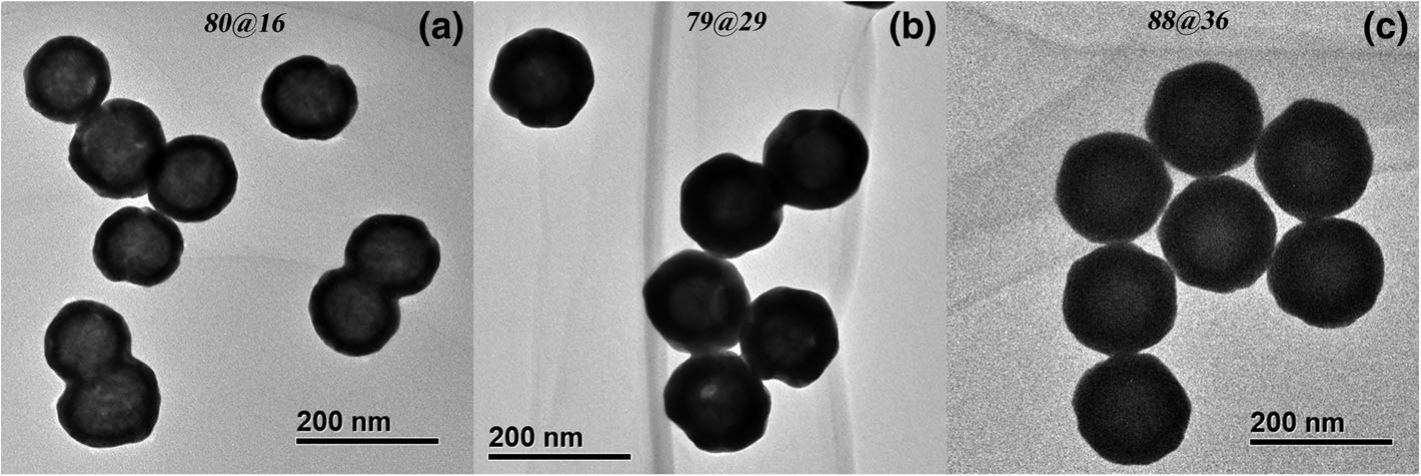
TEM images of the three gold nanoshells used in experimental measurements. **a** 80-nm-diameter silica core, 16-nm-thick gold shell. **b** 79-nm-diameter silica core, 29-nm-thick gold shell. **c** 88-nm-diameter silica core, 36-nm-thick gold shell. The detailed characterization information is provided in supporting information

**Fig. 4:**
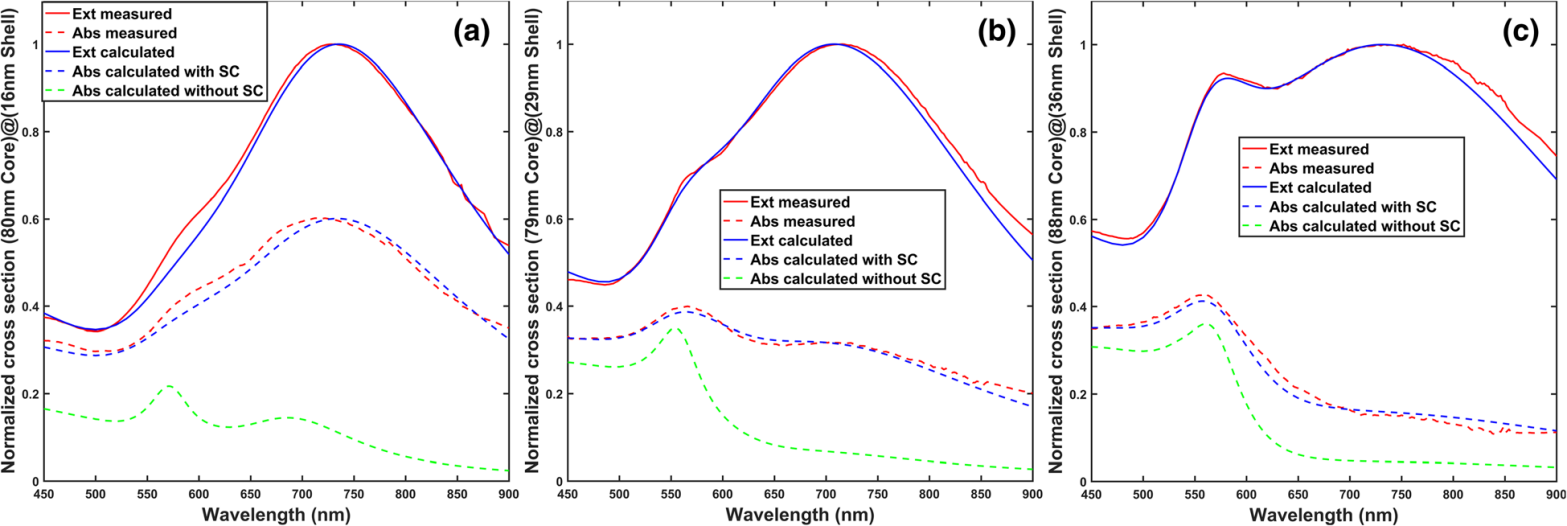
Comparisons between measured and calculated absorption spectrums with and without consideration of surface scattering of conduction electrons. All calculations are carried out by Mie theory. The surrounding medium is PVA (polyvinyl alcohol) having a refractive index of 1.5. Note that the surrounding medium of nanoshells in our experiment (PVA, *n* = 1.5) is different from that in the characterization sheet provided in the supporting information, which is water. The surfactants coating the nanoshells can be neglected because the surfactants are thin polymer and have similar refractive index as the surrounding medium of PVA. The refractive index of silica is assumed to be 1.45 in the calculations. Gold’s dielectric constants without surface scattering are from reference [38], while those with surface scattering are given by Eqs. (1) and (4). In all figure legends, “Ext” is short for extinction, “Abs” is short for absorption, and “SC” is short for surface scattering. In each figure, “Ext calculated” and “Abs calculated with SC” are the extinction and absorption spectrums calculated with the fitting parameters shown in Table 2, while “Abs calculated without SC” is the absorption cross section calculated without considering size distribution and surface scattering. **a** (80-nm-diameter silica core)@(16-nm-thick gold shell). **b** (79-nm-diameter silica core)@(29-nm-thick gold shell). **c** (88-nm-diameter silica core)@(36-nm-thick gold shell)

Subject to availability of material, absorption and extinction are experimentally measured for three nanoshells of different shell thicknesses but similar core diameters: (80-nm-diameter silica core)@(16-nm-thick gold shell), (79-nm-diameter silica core)@(29-nm-thick gold shell), and (88-nm-diameter silica core)@(36-nm-thick gold shell), whose TEM images are shown in Fig. 3. Figure 4 shows the comparison between the experimentally measured and theoretically simulated results for the three nanoshells. It can be observed in Fig. 4 that the calculated absorption cross sections with the surface scattering effect taken into account agree well with the measured results for all of the three nanoshells, while there is a large departure between the measured and simulated absorptions if the surface scattering effect is not considered.

To fit the calculated extinctions (the solid blue lines) to the experimentally measured extinctions (the solid red lines), which are shown in Fig. 4, the expression of the extra damping *γ*_s_ in Eq. (1) due to surface scattering is given by Eq. (4) shown below [15], instead of Eq. (2).

4 $$ {\gamma}_{\mathrm{s}}=\frac{A{v}_{\mathrm{F}}}{d_{\mathrm{s}}} $$

where *A* is a dimensionless fitting parameter and a larger *A* indicates a larger damping and *d*_s_ is the shell thickness. The fitting parameter *A* is affected by many factors: electron density at the surface, effect of the interface, anisotropy of particle, and quantum mechanical computation, and its value has been shown to range from 0.1 to above 2 [40, 41]. Note that we can write Eq. (2) into the form of Eq. (4) to compare the theoretical value of *A* predicted by billiard scattering model to those fitted from experiment, by first calculating the value of *L*_B_ in Eq. (2) using Eq. (3), and then writing *L*_B_ in Eq. (2) into the form of *d*_s_/*A*, as shown in Eq. (5a) to (5d) below:

5a $$ {\gamma}_{\mathrm{s}}=\frac{v_{\mathrm{F}}}{L_{\mathrm{B}}}={v}_{\mathrm{F}}\bullet \frac{3\left({r}_{\mathrm{o}}^2+{r}_{\mathrm{i}}^2\right)}{4\left({r}_{\mathrm{o}}^3-{r}_{\mathrm{i}}^3\right)} $$

write

5b $$ \frac{3\left({r}_{\mathrm{o}}^2+{r}_{\mathrm{i}}^2\right)}{4\left({r}_{\mathrm{o}}^3-{r}_{\mathrm{i}}^3\right)}=\frac{A}{d_{\mathrm{s}}} $$

then

5c $$ {\gamma}_{\mathrm{s}}=\frac{A{v}_{\mathrm{F}}}{d_{\mathrm{s}}} $$

where

5d $$ A={d}_{\mathrm{s}}\bullet \frac{3\left({r}_{\mathrm{o}}^2+{r}_{\mathrm{i}}^2\right)}{4\left({r}_{\mathrm{o}}^3-{r}_{\mathrm{i}}^3\right)} $$

Note that when the shell thickness is less than 25% of the total radius, Eq. (5d) gives an *A* value of about 0.5 [13]. The values of the fitting parameters for the calculated spectrums of the three nanoshells shown in Fig. 4 are tabulated in Table 2.

**Table 2:** Values of the fitting parameters for the calculated spectrums shown in Fig. 4

Structure	(80-nm-diameter silica core)@(16-nm-thick gold shell)	(79-nm-diameter silica core)@(29-nm-thick gold shell)	(88-nm-diameter silica core)@(36-nm-thick gold shell)
Core diameter (nm)	86	87	89
Shell thickness (nm)	13.5	25	35
*A*	1.33	1.67	1.33
Standard deviation of core diameter (nm)	4.0	3.4	6.0
Standard deviation of shell thickness (nm)	0.6	1.0	2.3

The calculated extinction and absorption spectrums shown in Fig. 4, which are normalized to the dipolar peak, have considered the surface scattering and size distribution. For each nanoshell, the standard deviations of the core diameter and shell thickness are computed respectively by multiplying the core diameter and shell thickness values shown in Table 2 with the coefficient of variation given in the characterization sheets provided in the supporting information. The core diameters used in the fitting are larger than the values given in the characterization sheets. This is because silica sphere’s size shrinks under TEM examination [42, 43], and the shell thicknesses are obtained by subtracting the core diameters in Table 2 from the total diameters given in characterization sheets. The calculated extinction spectrums’ peak widths are tuned to match the measured ones, and then, the corresponding absorption spectrums are calculated with the tuned parameters. The values of *A* predicted by billiard scattering model would be 0.60, 0.52, and 0.53 for the three nanoshells respectively if Eq. (5d) is applied, which are obviously smaller than the fitted *A* values listed in Table 2, which are 1.33, 1.67, and 1.33, respectively for the three nanoshells. Since a larger value of *A* in Eq. (4) means a larger damping of free electrons, it is observed that the actual damping of conduction electrons is larger than that predicted by the billiard scattering model, where the extra damping could be due to the chemical interface between the shell and the surrounding medium as well as the silica core [44, 45], electron density at the surface, anisotropy of particle, and quantum mechanical computation, as mentioned earlier. The possibility of discontinuous shell can be excluded by observing the TEM image in the characterization sheet in supporting information. Note that the peak broadening due to nanoshell’s size distribution has already been considered during the fitting, i.e., the fitted values of *A* does not account for size distribution. The details of how to measure the extinction and absorption are described in the section “Methods/Experimental.”

## Methods/Experimental

In this section, for the nanoshells studied in Fig 4, it is described how to disperse them into PVA (polyvinyl alcohol) thin films and how to derive the extinction and absorption of these nanoshells from optical measurements of the nanoparticle-dispersed PVA thin films.

The three nanoshells studied in Fig. 4, i.e., (80-nm-diameter silica core)@(16-nm-thick gold shell), (79-nm-diameter silica core)@(29-nm-thick gold shell), and (88-nm-diameter silica core)@(36-nm-thick gold shell), which for convenience are in abbreviation as 16-nm, 29-nm, and 36-nm gold nanoshells respectively in the following discussion, were purchased directly from a specialized company, nanoComposix, and their characterization sheets are shown in supporting information (Additional file 1).

**Fig. 5:**
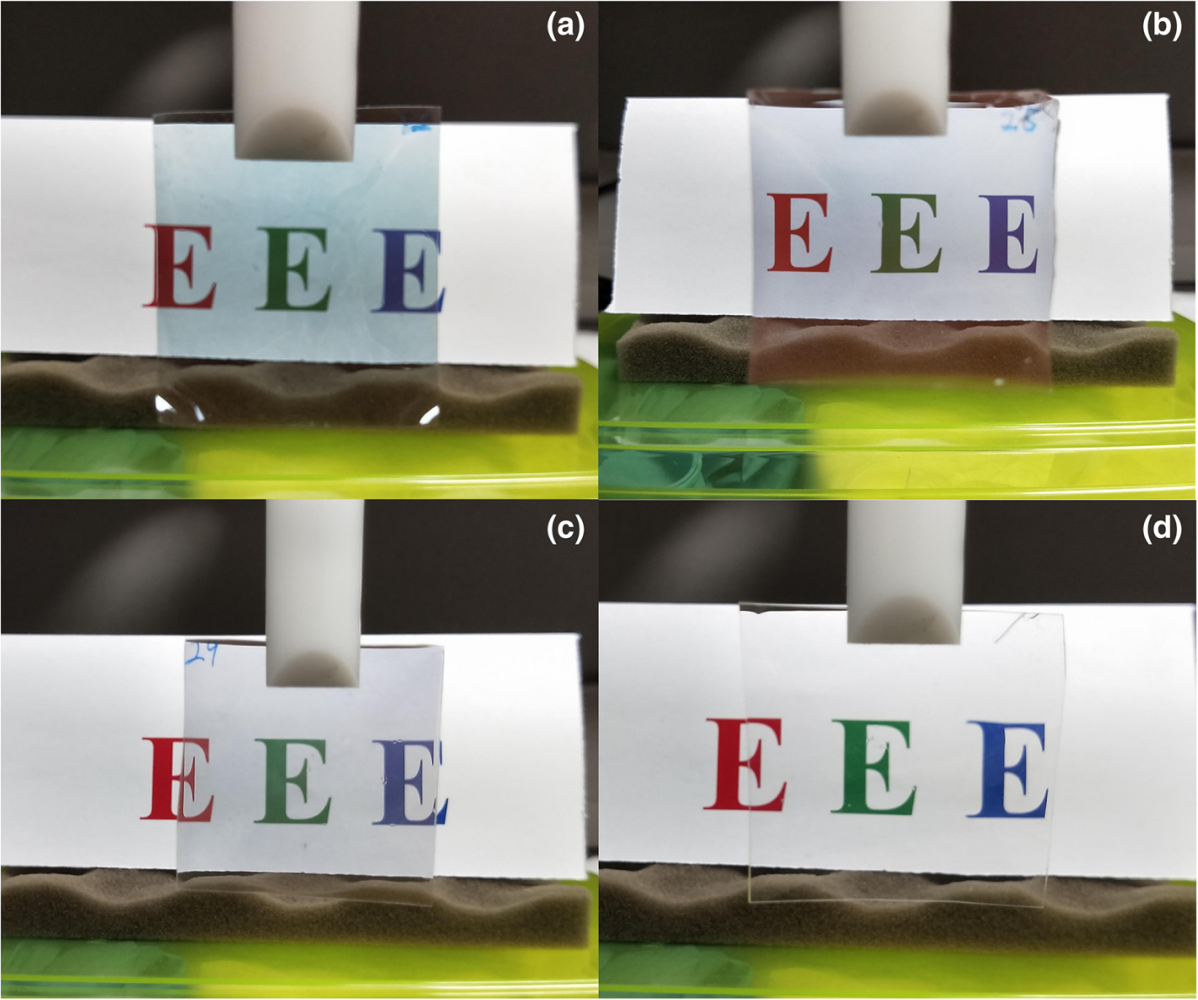
**a** Film dispersed with the 16-nm gold nanoshell. **b** Film dispersed with the 29-nm gold nanoshell. **c** Film dispersed with the 36-nm gold nanoshell. **d** Pure PVA film

The nanoshells were dispersed in water upon receiving, with the 16-nm gold nanoshell having a concentration of 0.02 mg/mL and the other two having a concentration of 0.05 mg/mL. For the 16-nm, 29-nm, and 36-nm gold nanoshells, 34, 25, and 34 mL of their solutions were used to make the nanoparticle-dispersed PVA film. Before mixing the as-received nanoshell solutions with PVA powder (80% hydrolyzed, Sigma-Aldrich), each nanoshell solution was concentrated to 9 mL by centrifugation and re-dispersion. And then 0.9-g PVA powder was added to each concentrated nanoshell solution, and the mixtures were stirred for 2 h. After this, each stirred solution was debubbled in a vacuum chamber and was then poured into a 5 × 5 cm^2^ glass mold, and the mold was put in a fume hood to let the solution naturally dry. After the solutions were dried, the PVA films were teared from the glass molds, and they are shown in Fig. 5. A pure PVA film without any nanoparticle dispersed was made likewise, except that 9 mL of water instead of nanoshell solution was mixed with PVA powder.

The extinction cross section *σ*_ext_ of a nanoshell is linked to direct transmittance *T* of a thin film of nanoshells through Beer-Lambert law [44]:

6 $$ T={e}^{-N\bullet {\sigma}_{\mathrm{ext}}} $$

where *N* is the areal density of nanoparticles, i.e., number of nanoshells per unit area (note that this area is perpendicular to the propagation direction of the incident light). The direct transmittance *T* is obtained by normalizing the measured direct transmittance of a PVA film dispersed with nanoshell to that of the pure PVA film without any nanoparticle dispersed. So *N* ∙ *σ*_ext_ is given by the following equation:

7 $$ N\bullet {\sigma}_{\mathrm{ext}}=-\ln (T) $$

Note that instead of *σ*_ext_, only *N* ∙ *σ*_ext_ is derived from experimental measurements, because it is the overall shape of the spectrum that matters. In Fig. 4, *N* ∙ *σ*_ext_ is normalized in a way such that the maximum value of *N* ∙ *σ*_ext_ of the spectrum is 1.

The absorption cross section *σ*_abs_ of a single nanoshell is related to the intensity loss of a parallel beam of incident light due to absorption *∆I*_abs_ after it passes through a thin film of nanoparticles, based on the Beer-Lambert law [44]:

8 $$ \Delta  {I}_{\mathrm{abs}}={I}_0\left(1-{e}^{-N\bullet {\sigma}_{\mathrm{abs}}}\right) $$

where *I*_0_ is the intensity of the incident light.

**Fig. 6:**
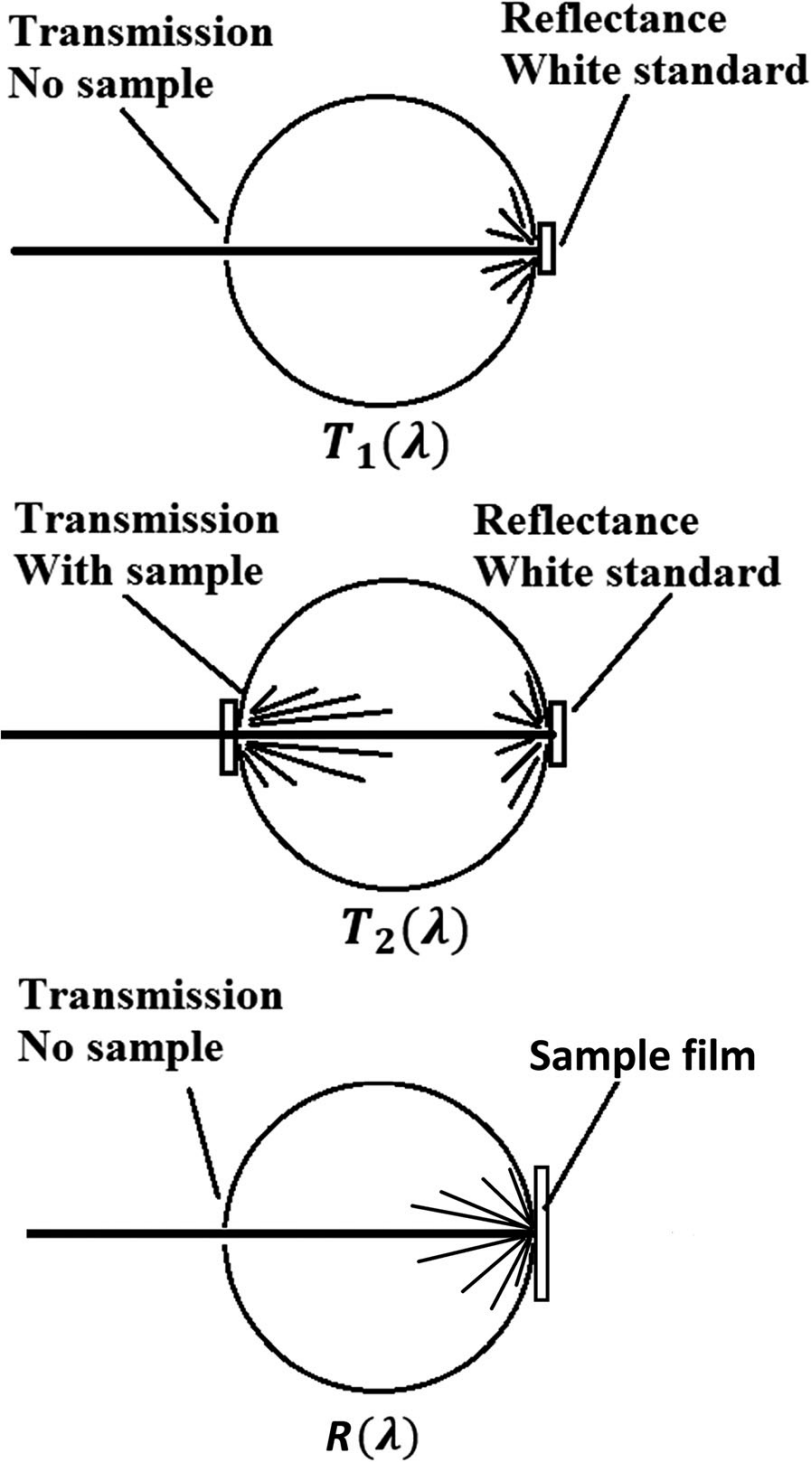
Experimental setup to measure absorption. The directly measured values are *T*_1_(*λ*), *T*_2_(*λ*), and *R*(*λ*) where *T*_*i*_ (*i* = 1, 2) or *R*(*λ*) is proportional to the amount of light trapped in the integrating sphere. The absorption is deduced from these measured values. This setup is a simplified version of the one reported in reference [22]

So, the next step is to experimentally find attenuation of the incident light only due to nanoparticles’ absorption. Equation (8) assumes the particle to be purely absorbing [44]. For nanoparticle that absorbs and scatters light simultaneously, Eq. (8) is not valid because of multiple absorptions. For an ensemble of such nanoparticles, when the incident light first hits a nanoparticle, some rays of the light are absorbed, and some are scattered. But for these scattered rays of light, when they hit more nanoparticles during their way out of the nanoparticles’ ensemble, a portion of them are absorbed again, leading to multiple absorptions. Multiple absorptions of the scattered light suggest that by measuring the total amount of light that is not absorbed by the PVA film dispersed with nanoshells, the *N* ∙ *σ*_abs_ derived according to Eq. (8) tends to overestimate absorption. However, since the PVA film in our experiments is thin (about 0.3 mm), and the nanoshells’ concentration is not high, it is assumed that most of the light undergoes single scattering (and thus single absorption) [25]. With this assumption, the experimental setup using an integrating sphere to measure total amount of light that is not absorbed by the PVA film dispersed with nanoshells is shown in Fig. 6. In Fig. 6 *T*_1_, *T*_2_, or *R* is proportional to the amount of light trapped in the integrating sphere, i.e., the amount of light that fails to go out from the open port at the right side. In the following discussion, it is assumed that *T*_1_, *T*_2_, and *R* are proportional to the light intensity collected by the integrating sphere with the same coefficient *α*.

Equation (8) can be rearranged into $\left({I}_0-\Delta  {I}_{\mathrm{abs}}\right)={I}_0{e}^{-N\bullet {\sigma}_{\mathrm{abs}}}$, and its left-hand side represents the total amount of light that is not absorbed after the incident light passes through the sample film. From the measurements in Fig. 6, we can write the following equations:

9 $$ \left({I}_0-\Delta  {I}_{\mathrm{abs}}\right)=\upalpha \left({T}_2\left(\lambda \right)+R\left(\lambda \right)\right) $$

10 $$ {I}_0=\upalpha {T}_1\left(\lambda \right) $$

Substituting Eq. (9) and Eq. (10) into $\left({I}_0-\Delta  {I}_{\mathrm{abs}}\right)={I}_0{e}^{-N\bullet {\sigma}_{\mathrm{abs}}}$, and including a noise term in addition to *N* ∙ *σ*_abs_, the following equation can be obtained:

11 $$ \frac{T_2\left(\lambda \right)+R\left(\lambda \right)}{T_1\left(\lambda \right)}={e}^{-\left(N\bullet {\sigma}_{\mathrm{abs}}+ Noise\right)} $$

where *Noise* is from the PVA matrix. Due to the first reflection of the incident light at the air/PVA interface, about 4% of incident light never enters the thin film (according to Fresnel equations, upon normal incidence at an interface of two different media of indices *n*_1_ (= 1 for air) and *n*_2_ (= 1.5 for PVA), the reflectance of light *R* is given by $R={\left|\frac{n_1-{n}_2}{n_1+{n}_2}\right|}^2$) and thus Eq. (11) is modified as

12 $$ \frac{T_2\left(\lambda \right)+R\left(\lambda \right)-0.04{T}_1\left(\lambda \right)}{T_1\left(\lambda \right)-0.04{T}_1\left(\lambda \right)}={e}^{-\left(N\bullet {\sigma}_{\mathrm{abs}}+ Noise\right)} $$

Assuming the *Noise* in the pure PVA film without any nanoparticle dispersed is the same as that in the nanoshell-dispersed films, a similar expression can be derived for the pure PVA film:

13 $$ \frac{T_2^{\prime}\left(\lambda \right)+{R}^{\prime}\left(\lambda \right)-0.04{T}_1\left(\lambda \right)}{T_1\left(\lambda \right)-0.04{T}_1\left(\lambda \right)}={e}^{- Noise} $$

where ${T}_2^{\prime}\left(\lambda \right)$ and *R*^′^(*λ*) are measured for the pure PVA film in the same way as *T*_2_(*λ*) and *R*(*λ*) for the nanoshell-dispersed film respectively.

From Eqs. (12) and (13), *N* ∙ *σ*_abs_ is given by the following expression:

14 $$ N\bullet {\sigma}_{\mathrm{abs}}=-\ln \left(\frac{T_2\left(\lambda \right)+R\left(\lambda \right)-0.04{T}_1\left(\lambda \right)}{T_2^{\prime}\left(\lambda \right)+{R}^{\prime}\left(\lambda \right)-0.04{T}_1\left(\lambda \right)}\right) $$

However, during the fitting to the experimental results, in which the value of *A* in Eq.(4) is adjusted such that the peak width of the calculated extinction spectrum fits the measured one, it is found that the normalized *N* ∙ *σ*_abs_ is still a little bit larger than the calculated absorption which includes the surface scattering effect. This suggests that multiple absorptions of scattered light may still contribute to extra absorption, as discussed previously. So, it is estimated here that a portion *p* (0 < *p* < 1) of the scattered light when no multiple absorptions happen is absorbed in the actual situation, where *p* is estimated to be 10% for the 16-nm nanoshell and 5% for both the 29 nm and 36 nm. The following two equations are set to account for the multiple scattering effect:

15 $$ N\bullet {\sigma_{\mathrm{abs}}}^{\prime }+N\bullet {\sigma_{\mathrm{sca}}}^{\prime }=N\bullet {\sigma}_{\mathrm{ext}} $$

16 $$ N\bullet {\sigma}_{\mathrm{abs}}+\left(1-p\right)N\bullet {\sigma_{\mathrm{sca}}}^{\prime }=N\bullet {\sigma}_{\mathrm{ext}} $$

where *N* ∙ *σ*_abs_^′^ and *N* ∙ *σ*_sca_^′^ are the light absorption and scattering, respectively, when no multiple absorptions happen, and *N* ∙ *σ*_abs_ and *N* ∙ *σ*_ext_ are the experimentally measured absorption and extinction given by Eq. (14) and Eq. (7) respectively. The extinction in Eqs. (15) and (16) is the same because multiple scattering does not induce error in the measurement of *N* ∙ *σ*_ext_. From Eqs. (15) and (16), the corrected expression for the measured absorption is given below:

17 $$ N\bullet {\sigma_{\mathrm{abs}}}^{\prime }=N\bullet {\sigma}_{\mathrm{ext}}-\frac{1}{\left(1-p\right)}\left(N\bullet {\sigma}_{\mathrm{ext}}-N\bullet {\sigma}_{\mathrm{abs}}\right) $$

In Fig. 4, the corrected absorption *N* ∙ *σ*_abs_^′^ is also normalized to the maximum value of the *N* ∙ *σ*_ext_ spectrum calculated with Eq. (7).

## Conclusions

In this work, surface scattering of conduction electrons in gold nanoshell is shown to not only broaden the extinction peak width, but also increase the ratio of light absorption to extinction and thus decrease the ratio of light scattering to extinction. It is also found that the thinner the shell thickness, the more increase of the absorption ratio. And the increase of light absorption ratio is verified by fitting of calculated absorption spectra to measured ones.

## Additional File

Additional file 1: Characterization sheets provided by nanoComposix for the 3 nanoshells used in the experiment. (PDF 2936 kb)
