# Supplementary material for: Effect of Surface Scattering of Electrons on Ratios of Optical Absorption and Scattering to Extinction of Gold Nanoshell
Source: Nanoscale Res Lett. 2018 Sep 25;13:299. doi: 10.1186/s11671-018-2670-7 (PMC6156710; doi:10.1186/s11671-018-2670-7)
Supplement: Supplementary file 1 — Characterization sheets provided by nanoComposix for the 3 nanoshells used in the experiment. (PDF 2936 kb) [file 11671_2018_2670_MOESM1_ESM.pdf]

80 nm Silica Core with 15 nm Gold Nanoshell, PVP

Lot Number: JAG0399

**Total Diameter (TEM):**	113 ± 5 nm	**Mass Concentration (Au):**	0.020 mg/mL
**Coefficient of Variation:**	4.6 %	**Hydrodynamic Diameter:**	144 nm
**Core Diameter (TEM):**	80 ± 6 nm	**Zeta Potential:**	-35 mV
**Shell Thickness (Calc'd):**	16 nm	**pH of Solution:**	5.6
**Surface Area (Calc'd):**	4.0 m^2/g	**Particle Surface:**	PVP
**Particle Concentration:**	2.1E+09 particles/mL	**Solvent:**	Milli-Q Water

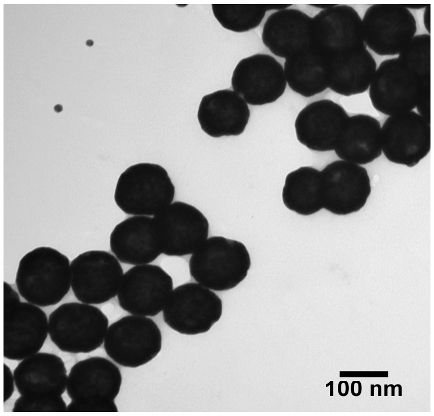

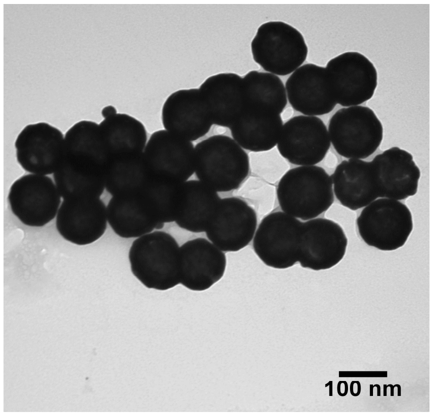

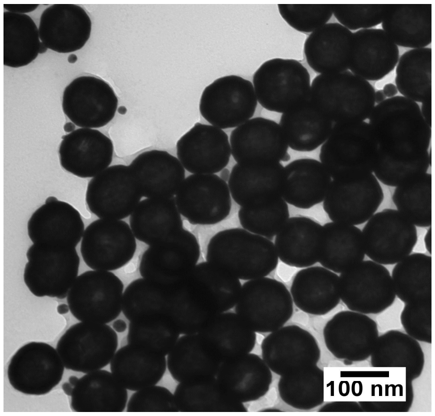

Size Distribution

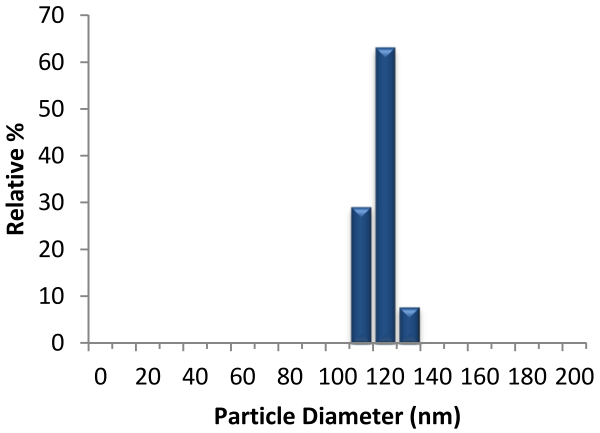

Optical Properties

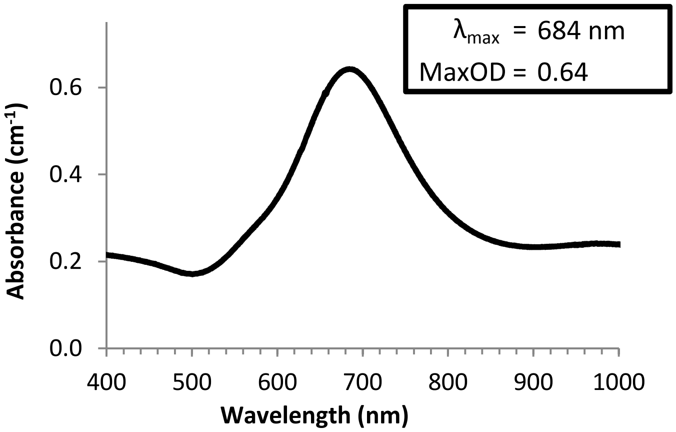

Characterization Instrumentation

**Diameter and Size Statistics:**	JEOL 1010 Transmission Electron Microscope
**Mass Concentration:**	Thermo Fisher X Series 2 ICP-MS
**Spectral Properties:**	Agilent 8453 UV-Visible Spectrometer
**Hydrodynamic Diameter/Zeta Potential:**	Malvern Zetasizer Nano ZS.

Shake vigorously before use. Bath sonicate if needed. Storage: 2-8 ^{o}C. DO NOT FREEZE.

Produced under license to United States patent numbers 6,344,272, 6,685,986, and 7,371,457.

These nanoComposix products are to be used for research purposes only and are not to be used for any commercial purposes.

nanoComposix, Inc
4878 Ronson Ct. Suite K
San Diego, CA 92111

nanoComposix.com

info@nanocomposix.com
Phone: (858) 565-4227
Fax: (619) 330-2556

Gold Nanoshells, Peak Absorbance @ 660 nm, PVP, NanoXact™

Lot Number: JRC0466

**Total Diameter (TEM):**	137 ± 5 nm	**Mass Concentration (Au):**	0.050 mg/mL
**Coefficient of Variation:**	3.9 %	**Hydrodynamic Diameter:**	157 nm
**Core Diameter (TEM):**	79 ± 3 nm	**Zeta Potential:**	-31 mV
**Shell Thickness (Calc'd):**	29 nm	**pH of Solution:**	5.9
**Surface Area (Calc'd):**	2.7 m^2/g	**Particle Surface:**	PVP 40 kDa (Polymer)
**Particle Concentration (Calc'd):**	2.4E+09 particles/mL	**Solvent:**	Milli-Q Water

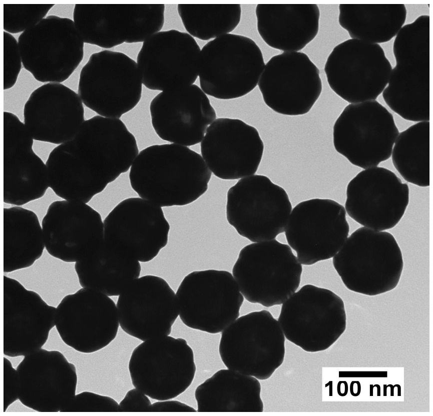

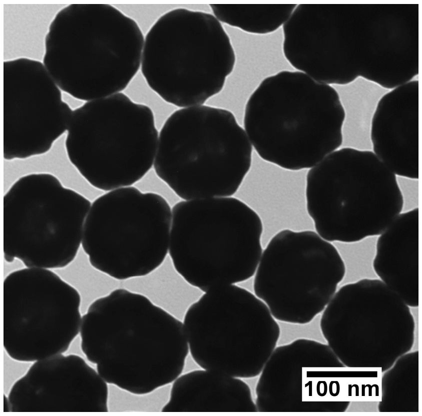

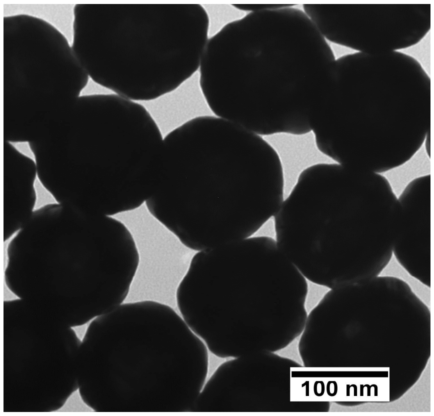

Size Distribution

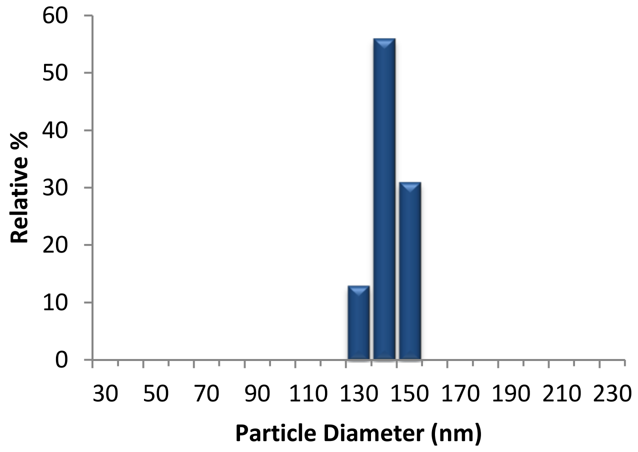

Optical Properties

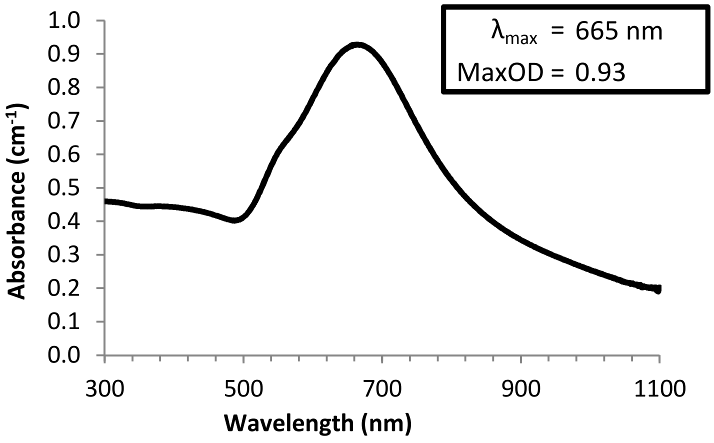

Characterization Instrumentation

**Diameter and Size Statistics:**	JEOL 1010 Transmission Electron Microscope
**Mass Concentration:**	Thermo Fisher X Series 2 ICP-MS
**Spectral Properties:**	Agilent 8453 UV-Visible Spectrometer
**Hydrodynamic Diameter/Zeta Potential:**	Malvern Zetasizer Nano ZS
**pH:**	Horiba - Laqua Twin pH Meter

Shake vigorously before use. Bath sonicate if needed. Storage: 2-8 ^oC. DO NOT FREEZE.

Produced under license to United States patent numbers 6,344,272, 6,685,986, and 7,371,457.

These nanoComposix products are to be used for research purposes only and are not to be used for any commercial purposes.

nanoComposix, Inc
4878 Ronson Ct. Suite K
San Diego, CA 92111

nanoComposix.com

info@nanocomposix.com
Phone: (858) 565-4227
Fax: (619) 330-2556

Gold Nanoshells, Peak Absorbance @ 660 nm, PEG, NanoXact™

Lot Number: JRC0398

**Total Diameter (TEM):**	159 ± 11 nm	**Mass Concentration (Au):**	0.051 mg/mL
**Coefficient of Variation:**	6.7 %	**Hydrodynamic Diameter:**	181 nm
**Core Diameter (TEM):**	88 ± 3 nm	**Zeta Potential:**	-28 mV
**Shell Thickness (Calc'd):**	36 nm	**pH of Solution:**	5.5
**Surface Area (Calc'd):**	2.3 m^2/g	**Particle Surface:**	mPEG 5 kDa (Polymer)
**Particle Concentration (Calc'd):**	1.5E+09 particles/mL	**Solvent:**	Milli-Q Water

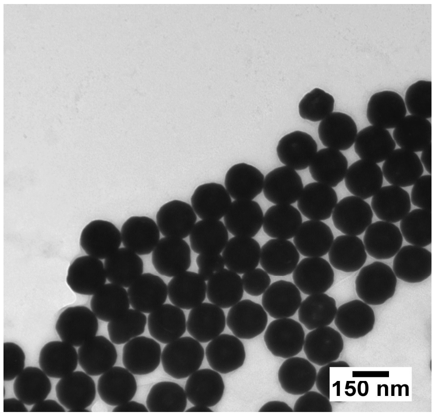

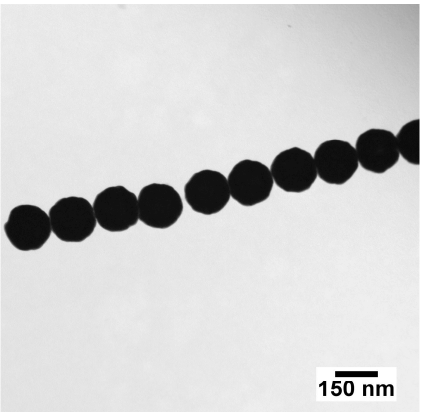

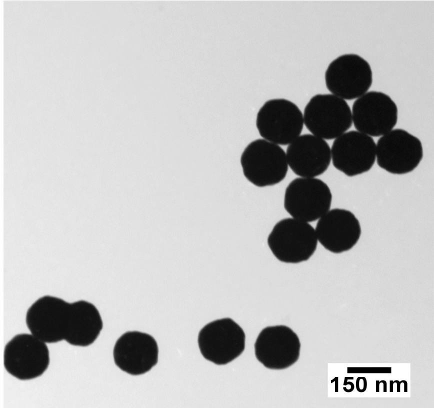

Size Distribution

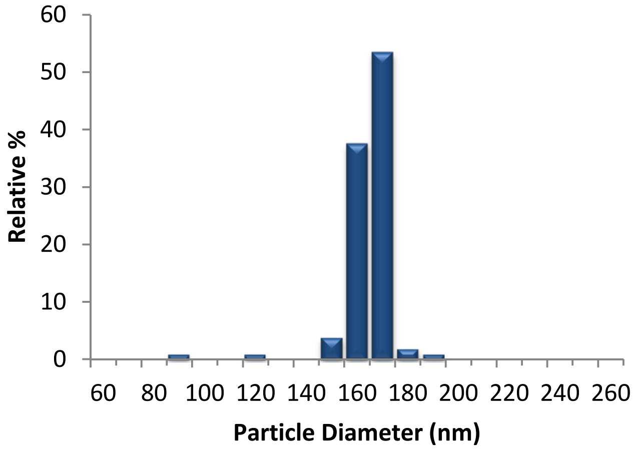

Optical Properties

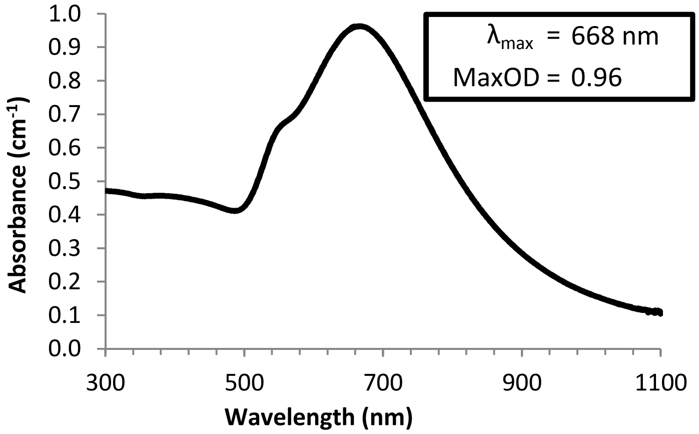

Characterization Instrumentation

**Diameter and Size Statistics:**	JEOL 1010 Transmission Electron Microscope
**Mass Concentration:**	Thermo Fisher X Series 2 ICP-MS
**Spectral Properties:**	Agilent 8453 UV-Visible Spectrometer
**Hydrodynamic Diameter/Zeta Potential:**	Malvern Zetasizer Nano ZS
**pH:**	Horiba - Laqua Twin pH Meter

Shake vigorously before use. Bath sonicate if needed. Storage: 2-8 ^oC. DO NOT FREEZE.

Produced under license to United States patent numbers 6,344,272, 6,685,986, and 7,371,457.

These nanoComposix products are to be used for research purposes only and are not to be used for any commercial purposes.

nanoComposix, Inc
4878 Ronson Ct. Suite K
San Diego, CA 92111

nanoComposix.com

info@nanocomposix.com
Phone: (858) 565-4227
Fax: (619) 330-2556
